# Anemia and hemoglobin serum levels are associated with exercise capacity and quality of life in chronic obstructive pulmonary disease

**DOI:** 10.1186/s12890-015-0050-y

**Published:** 2015-05-08

**Authors:** Marcello Ferrari, Lorenzo Manea, Kamel Anton, Paola Bruzzone, Mara Meneghello, Francesco Zamboni, Luigi Purgato, Lucia Cazzoletti, Pietro Ferrari, Renato Testi

**Affiliations:** Unit of Respiratory Diseases, Department of Medicine, University of Verona, Verona, Italy; School of Sports Medicine, University of Padova, Verona, Trieste and Udine Italy; Unit of Epidemiology and Medical Statistics, Department of Public Health and Community Medicine, University of Verona, Verona, Italy; Servizio di Fisiopatologia Respiratoria, Policlinico G.B.Rossi, 37121 Verona, Italy

**Keywords:** COPD, Exercise capacity, Hemoglobin, Muscular strenght, Quality of life

## Abstract

**Background:**

Little is known about the relationship between hemoglobin concentrations, functional status and health related quality of life (HRQL) in chronic obstructive pulmonary disease (COPD). Our aim was to investigate the prevalence of anemia and the association of hemoglobin with shortness of breath, exercise capacity, muscle strength and HRQL, in COPD patients.

**Methods:**

A total of 105 COPD patients (77 males, 71.6 ± 9.2 years) were studied. Patients were classified as anemic and non anemic using the WHO criteria. We used the Medical Research Council Dyspnoea scale (MRCs) to measure shortness of breath. Exercise capacity was assessed using the six minute walking distance (6MWD) and the peak of VO2 during the maximal cycle ergometer test (VO2_max_). We used the Quadriceps and Handgrip strength assessment to determine muscle strength. The Saint George Respiratory Questionnaire was used to investigate HRQL. The physiological/functional characteristics of the two groups were compared. Regression models adjusting for confounders examined the independent association of anemia and of hemoglobin levels with clinical and functional outcomes.

**Results:**

Anemic patients (12.3%) showed a significantly higher MRCs, a lower 6MWD, VO2_max_, and a worse quality of life. On the contrary, there was no difference in muscle strength between the two groups. In the regression models, hemoglobin was independently associated with reduced exercise capacity and HRQL.

**Conclusions:**

Anemia in COPD was a risk factor for poorer exercise capacity and quality of life, and these outcomes were linearly associated with hemoglobin. Our results should stimulate further research into exploring whether increasing hemoglobin has a beneficial effect on the outcomes in COPD.

## Background

Chronic obstructive pulmonary disease (COPD), a highly prevalent condition associated with increased morbidity and mortality, is basically characterized by the presence of expiratory flow limitation that is not fully reversible [1]. Great importance has recently been given to the role of inflammation in this disease, which could explain some of the extra-pulmonary manifestations of COPD, such as weight loss, muscle atrophy and the consequent reduction in exercise capacity [2-4]. Some authors [5] consider that anemia is also caused by the inflammatory process.

Anemia is frequently concomitant with many chronic diseases and is associated with increased morbidity and mortality [6-14]. Anemia is also characterized by a feeling of weakness and fatigue, and it has been suggested that it may contribute to dyspnoea and exercise limitation in patients with chronic diseases and also when this condition affects the elderly [15,16].

Several previous studies have evaluated the prevalence of anemia in chronic obstructive pulmonary disease [5,17,18]. On the contrary, scarce research has focused on examining the relationship between hemoglobin concentrations, dyspnoea, and exercise capacity [5,17] in COPD, and no study has been performed on its correlation with muscle strength. It is worth noting that only one investigation has been carried out on the relationship between hemoglobin levels and HRQL, which is an important clinical outcome in patients with chronic lung disease [19].

The aims of the study were to assess in stable COPD patients: 1) the distribution of hemoglobin values, the prevalence of anemia and their relationship with shortness of breath, exercise capacity and muscle strength; 2) the association between hemoglobin levels, anemia and HRQL.

## Methods

The present study was a retrospective analysis of the data collected in the medical records of the COPD patients attending our outpatient clinic from January 2013 to December 2013. The review board of the Department of Medicine of Verona University approved the access to patient records and patient confidentiality was maintained.

The patients were included in the study if they satisfied the following criteria: a diagnosis of COPD [20] and a stable clinical condition, which meant that they had received continuous treatment and had had no exacerbation during the previous two months. The exclusion criteria were a severe cardiovascular disease, malignant diseases, systemic rheumatologic or connective tissue disorders, chronic renal failure. Additional exclusion criteria were the use of oral corticosteroid and long term oxygen therapy, and the inability to perform the exercise tests.

### Clinical assessment and anthropometric measurements

Smoking habits and the presence of comorbid conditions was determined by using self-reported history, medical examination data and medical records information. Scores on the Charlson comorbidity index (CCI), a method for classifying comorbid conditions, were calculated [21] from these data. Functional dyspnoea was measured using the Medical Research Council dyspnoea scale (MRCs) [22]. BODE index, a composite index of COPD severity, was also calculated [23]. In order to evaluate Health Related Quality of Life (HRQL), patients filled in the St. George’s Respiratory Questionnaire (SGRQ). Body mass index (BMI) was obtained by dividing body weight by height squared (kg/m2).

On the same day of the clinical assessment, each patient underwent a peripheral venous blood sampling, a pulmonary function testing, an evaluation of muscle strength and 6MWT. Exercise testing was performed on a separate day, within a week from the beginning of clinical assessment.

### Blood sample and analysis

All the patients underwent peripheral venous blood sampling to determine haemoglobin (Hb) concentrations (hematology autoanalyzer Advia 2120i, Simens Healthcare, Milan) and C-reactive protein concentrations (CRP) (immunoturbidimetric assay, Cobas 6000, Roche Diagnostics, Milan). Anemia was defined by using the WHO criteria, which meant that the Hb concentration was below 12 g/dL in women and below 13 g/dL in men [24]. Patients were categorized as polycythemic when they presented Hb levels ≥ 17 g/dL and ≥15 g/dL in males and females, respectively [5].

### Pulmonary function test

Spirometry was done pre and post use of bronchodilator (salbutamol 400mcg) and forced expiratory volume in 1-second (FEV1), forced vital capacity (FVC), FEV1/FVC were measured in accordance with ATS/ERS guidelines [25] by using a Sensor Medics Spirometer (Jorba Linda, Ca, USA). Predicted values were calculated according to Quanier [26].

### Muscle strength

Handgrip strength was measured in kg using a hydraulic hand-held dynamometer (Saehan Corp., Masan, Korea). The participants were asked to perform the task three times with each hand. The average of the best results obtained with each hand was used for the analysis. Isometric knee extension strength (kg) was measured in both legs with a dynamometer (Kern CH50K50, Kern & Sohn, Balingen, Germany), which was applied with a strap around the ankle, just proximal to the malleolli. After one try out, the best of three measurements was recorded on both sides. The average of the best measurement obtained on each side was used in the analysis.

### Exercise testing

The 6-min walk distance (6MWD) was performed according to the ATS statement [27]. Patients were instructed to walk as far as possible for 6 minutes, stopping for a rest if necessary. The total distance walked was measured to the nearest meter and recorded.

All the subjects performed a maximal bicycle test until exhaustion, according to the criteria of ATS on cardiopulmonary exercise testing [25]. The subjects cycled on an electrically braked cycle ergometer (Corival 400, Lode, Groningen, The Netherlands) at a pedalling rate of 60 rpm, breathing room air. After unloaded pedalling for 3 minutes, the workload was increased every minute by 5–20 W, until exhaustion. Exercise parameters and VO2 (L/min) were collected breath-by-breath and averaged over 10-second intervals, using ZAN600 cardiopulmonary measuring device (ZAN600 Ferraris, Germany).

### Statistical analysis

The between-group differences in baseline characteristics of COPD, with and without anemia, were expressed as mean ± sd values and these differences were calculated by using a two-sided test for independent samples, or Chi-squared statistic where appropriate. A p-value <0.05 was considered statistically significant. Linear regression analysis controlling for age, sex, BMI, smoking habits, PCR serum levels, FEV1, CCI, were used to evaluate the independent association of anemia with dyspnoea (MRCs), physical performance (6MWD, VO2_max_, Handgrip and Quadriceps strength) and quality of life (SGRQ). In similar models, the association of hemoglobin concentrations with the same outcomes was evaluated. The data analysis was performed using the Statistical Package for Social Science, version 20.0 (SPSS, Chicago, IL).

## Results

Out of the 105 patients included in the study, 13 (12.3%) had anemia, while polycythemia was recorded in 7 (6.7%) patients. The anemia was mild in most cases, with a mean hemoglobin value of 11.9 ± 0.5 g/dL. Table 1 shows the characteristics of the patients in our series and the differences in clinical data between the groups of cases with or without anemia. Patients in the anemia group had more severe COPD in terms of FEV1 (p < 0.0001), either when expressed in absolute or in percent of predicted values. Also the BODE index was higher in anemic subjects (p = 0.001). The mean CRP plasma concentrations were higher in anemic patients with respect to those without anemia, although the difference was not statistically significant, which was probably due to the high standard deviation of our data. There were no differences in age, sex, BMI, smoking history and CCI between patients with or without anemia. A significant negative relationship was found between CRP serum concentrations and hemoglobin levels (r = −0.349, p < 0.0001, data not shown) in a bivariate analysis.

**Table 1:** **Clinical characteristics of COPD patients divided into two groups on the basis of the presence of anemia**

**Variable**	**Total**	**Anemic**	**Non anemic**	p-value*
**Subjects ( n)**	105	13 (12.4)	92 (87.6)	
**Male sex (n(%))**	77 (73)	11 (85)	66 (71.7)	0.326
**Age (yrs)**	71.6 ± 9.2	72.8 ± 8.7	71.4 ± 9.3	0.606
**Weight (kg)**	74.6 ± 14.1	72.4 ± 14.2	74.9 ± 14.1	0.554
**Height (cm)**	164.1 ± 7.8	161.1 ± 7.0	164.6 ± 7.9	0.118
**BMI**	27.7 ± 4.6	27.8 ± 4.4	27.7 ± 4.6	0.968
**Smoking habits (Pack/year)**	39.0 ± 30.1	49.3 ± 38.8	37.7 ± 28.8	0.358
**VC (l)**	2.8 ± 0.8	2.6 ± 0.6	2.8 ± 0.8	0.361
**VC %predicted**	91.0 ± 22.1	85.3 ± 19.9	91.9 ± 22.4	0.294
**FEV1 (l)**	1.3 ± 0.6	0.9 ± 0.2	1.4 ± 0.6	**<0.0001**
**FEV1 % predicted**	55.7 ± 20.2	42.8 ± 9.7	57.6 ± 20.7	**<0.0001**
**BODE Index**	2.9 ± 2.3	5.0 ± 2.0	2.7 ± 2.2	**0.001**
**CCI**	2.5 ± 1.5	2.5 ± 2.0	2.5 ± 1.4	0.992
**Hemoglobin (g/dL)**	14.2 ± 1.3	11.9 ± 0.5	14.5 ± 1.1	**<0.0001**
**CRP (mg/L)**	13.4 ± 13.8	18.3 ± 14	12.8 ± 13.7	0.200

The data are presented as mean ± sd, unless otherwise stated. BMI: Body Mass Index; FEV_1_: Forced expiratory volume in 1 second; VC: Vital Capacity; BODE: Body mass index, airflow Obstruction, Dyspnoea, Exercise capacity, CCI: Charlson Comorbidity Index; CRP: C-reactive Protein.

* = calculated using 2 site unpaired t-tests.

Performance and strength outcomes, dyspnoea, and quality of life according to the presence or absence of anemia, are reported in Table 2. Mean MRCs values were significantly higher (2.8 ± 1.1 versus 1.6 ± 1.3; p = 0.002), mean 6MWD was significantly shorter (267.9 ± 86.7 versus 373.0 ± 122.8 m; p = 0.001), and VO2_max_ results lower (0.9 ± 0.2 versus 1.2 ± 0.4 L/min; p = 0.011) in anemic compared with non anemic patients. On the contrary, the mean values of handgrip (28.7 ± 6.5 versus 29.8 ± 8.5 kg) and quadriceps strength (21.3 ± 5.1 versus 21.0 ± 7.7 kg) were similar in the two groups. Quality of life, measured by using the SGRQ questionnaire, was significantly worse in subjects with anemia, as far as either total score or symptoms, activity or impact scores were concerned.

**Table 2:** **Exercise capacity, muscular strength, degree of dyspnoea and quality of life in COPD patients, divided according to the presence of anemia**

	**Total**	**Anemic**	**Non anemic**	p-value*
**6MWD (m)**	360.0 ± 123.6	267.9 ± 86.7	373.0 ± 122.8	**0.001**
**VO2**_**max**_ **(l/min)**	1.2 ± 0.4	0.9 ± 0.2	1.2 ± 0.4	**0.011**
**Handgrip (Kg)**	29.7 ± 8.3	28.7 ± 6.5	29.8 ± 8.5	0.572
**Quadriceps Strength (Kg)**	21.1 ± 7.4	21.3 ± 5.1	21.0 ± 7.7	0.853
**MRC Dyspnoea scale**	1.8 ± 1.4	2.8 ± 1.1	1.6 ± 1.3	**0.002**
**SGRQ Symptoms**	47.3 ± 23.3	63.6 ± 20.0	45.3 ± 22.9	**0.020**
**SGRQ Activity**	54.6 ± 24.3	78.6 ± 15.9	51.6 ± 23.5	**<0.0001**
**SGRQ Impact**	32.0 ± 21.2	53.4 ± 25.7	29.4 ± 19.1	**0.016**
**SGRQ Total**	41.4 ± 20.2	62.8 ± 20.9	38.7 ± 18.6	**0.005**

The data are presented as mean ± sd; 6MWD: six minutes walking distance; VO2_max_: maximal oxygen consumption; MRC: Medical Research Council; SGRQ: St George Respiratory Questionnaire.

* = calculated using 2 site unpaired t-tests.

**Figure 1:**
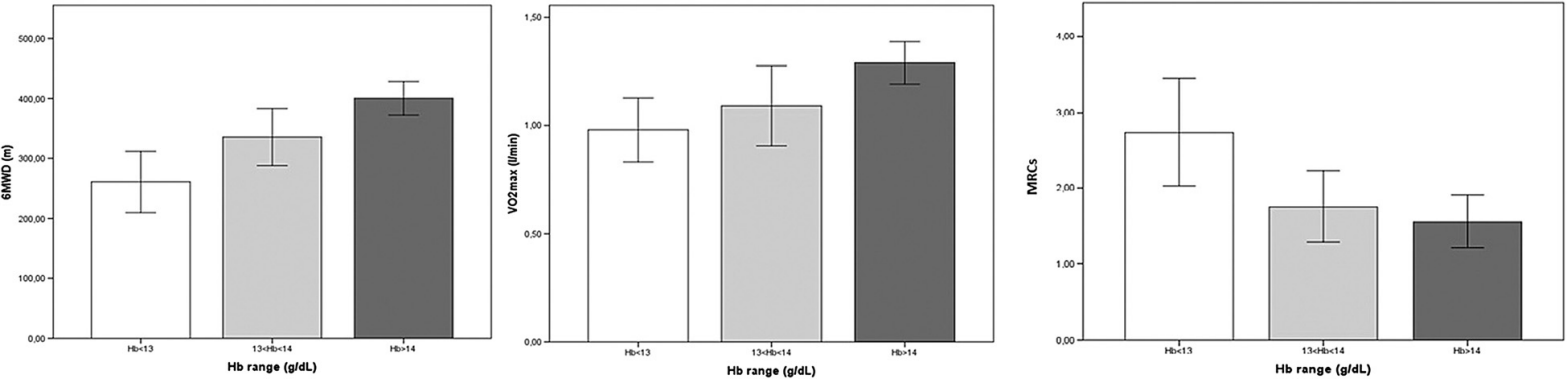
Hemoglobin and exercise capacity. Relationship between hemoglobin (Hb) level and six Minutes Walking Distance (6MWD), maximal oxygen consumption (VO2_max_) and Medical Research Council dysnoea scale (MRCs).

**Figure 2:**
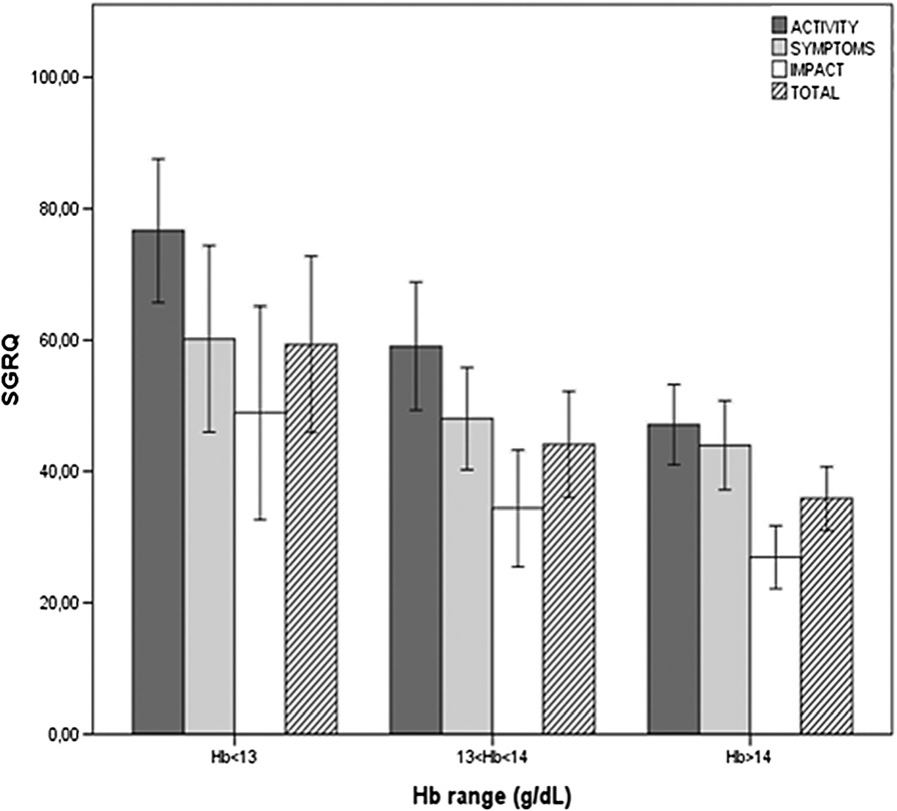
Hemoglobin and quality of life. Relationship between hemoglobin (Hb) level and the St George Respiratory Questionnaire (SGRQ) total **(**  **)**, activity **(**  **)**, symptoms **(**  **)** and impact **(**  **)** scores.

**Figure 3:**
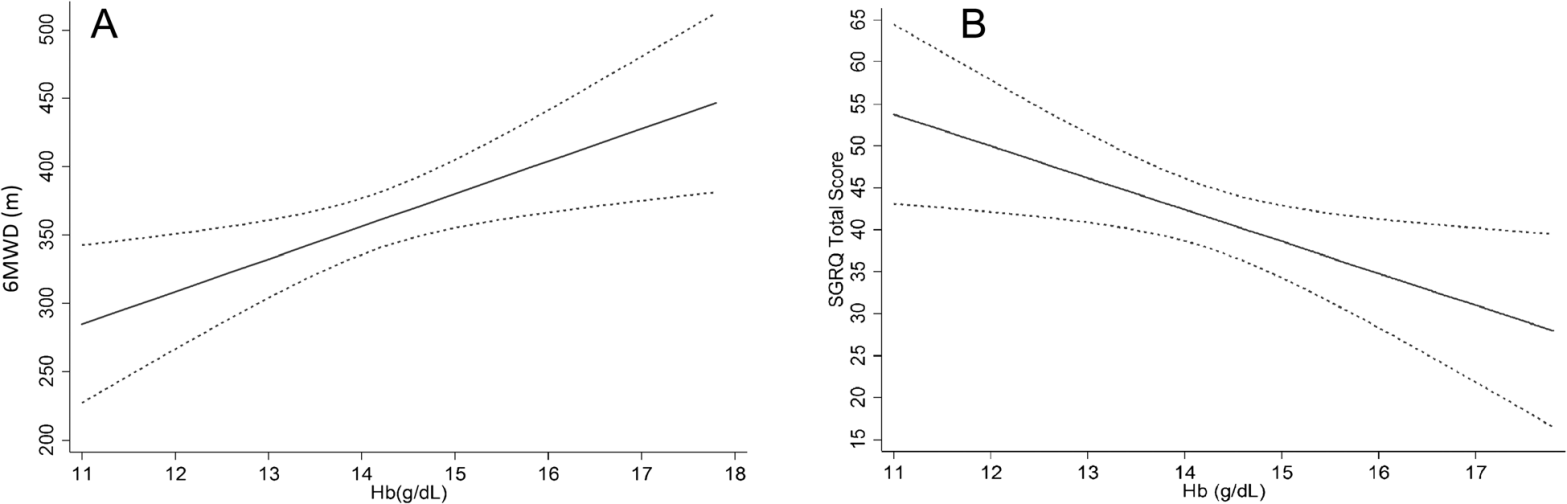
Relationship between hemoglobin, six Minutes Walking Distance and St George Respiratory Questionnaire. Results of a multivariate analysis of the relationship between hemoglobin (Hb) level and six Minutes Walking Distance (6MWD) **(panel A)** and the St George Respiratory Questionnaire (SGRQ) total score **(panel B)**. The predicted values of the 6MWT and SGRQ score were obtained by a regression model adjusted for sex, age, Body Mass Index (BMI), smoking habits, FEV1, the Charlson’s Comorbidity Index and the serum concentration of C-reactive Protein.

When MRCs, 6MWD, VO2_max_ and SGRQ scores were evaluated as a function of hemoglobin ranges, a linear relationship between declining hemoglobin on one hand, and increasing dyspnoea, worsening of exercise capacity and of quality of life on the other hand, was found (Figures 1 and 2). In regression models controlling for age, sex, BMI, FEV1, smoking habits (packyears), CCI and CRP, Hb levels remained an independent predictor of 6MWD (Table 3, Figure 3-panel A) and VO2_max_ (Table 3), but it was not associated with quadriceps strength and handgrip (data not shown). Furthermore, higher hemoglobin levels were correlated to a higher quality of life (Table 4, Figure 3-panel B).

**Table 3:** **Linear regression for the association among hemoglobin, exercise capacity and the degree of dyspnoea**

	**6MWD test (m)**				**VO2**_**max**_ **(l/min)**		**MRC dyspnoea scale**		
	**β**	**SE**	**p-value**	**β**	**SE**	**p-value**	**β**	**SE**	**p-value**
**Age**	−3.911	1.202	**0.002**	−0.013	0.004	**0.001**	0.012	0.015	0.416
**Sex**	−38.840	24.957	0.123	−0.467	0.080	**<0.001**	0.589	0.304	0.056
**BMI**	−8.791	2.545	**0.001**	0.019	0.008	**0.021**	0.070	0.031	**0.026**
**Smoking habits (pack/years)**	4.452	22.221	0.842	0.046	0.071	0.517	−0.056	0.271	0.837
**CCI**	−3.988	7.412	0.592	−0.012	0.024	0.607	0.151	0.090	0.099
**CRP (mg/L)**	0.859	0.862	0.322	0.001	0.003	0.667	−0.010	0.011	0.365
**FEV1%pred**	1.731	0.558	**0.003**	0.004	0.002	**0.019**	−0.021	0.007	**0.002**
**Hb (g/dL)**	24.108	8.693	**0.007**	0.080	0.028	**0.005**	−0.104	0.106	0.328

CCI: Charlson Comorbidity Index; CRP: C-reactive Protein; 6MWD: six minutes walking distance; VO2_max_: maximal oxygen consumption; MRC: Medical Research Council; β: beta-coefficent.

**Table 4:** **Linear regression for the association among hemoglobin and St. George Respiratory Questionnaire (SGRQ) activity, impact and total scores**

	**SGRQ activity**	**SGRQ impact**	**SGRQ total**						
	**β**	**SE**	**p-value**	**β**	**SE**	**p-value**	**β**	**SE**	**p-value**
**Age**	0.302	0.250	0.231	0.073	0.234	0.755	0.095	0.215	0.661
**Sex**	10.565	5.415	0.054	−1.419	5.074	0.780	2.341	4.659	0.617
**BMI**	1.092	0.548	**0.050**	−0.087	0.514	0.866	0.422	0.472	0.374
**Smoking habits (pack/years)**	−10.278	4.439	**0.023**	−3.880	4.160	0.354	−4.266	3.820	0.267
**CCI**	−1.523	1.591	0.341	0.619	1.491	0.679	−0.218	1.369	0.874
**CRP (mg/L)**	0.054	0.204	0.791	0.208	0.191	0.279	0.188	0.175	0.287
**FEV1%pred**	−0.367	0.119	**0.003**	−0.328	0.112	**0.004**	−0.352	0.103	**0.001**
**Hb (g/dL)**	−5.274	1.868	**0.006**	−3.443	1.751	**0.050**	−3.783	1.608	**0.021**

CCI: Charlson Comorbidity Index; CRP: C-reactive Protein.

## Discussion

The prevalence of anemia in this study (12.3%) is comparable to that found by John et al. (13%) [28] in subjects with COPD, and to that reported by Penninx et al. in Italy, in non-institutionalized elderly subjects (11.3%) [16]. The agreement with the data reported by Penninx et al. [16] indicates that the frequency of anemia in COPD is not greater than what was found in the general population. On the contrary, the prevalence we found in our study is less than that observed by Cote et al. in a cohort of patients with stable COPD (17%) [5] and by Halpern et al. (21%) [18]. The criteria used to identify anemia by Cote et al.( <13 g/dL independently from gender ) [5], and the difference in the population studied (subjects with COPD from the US Medicare Claims Database, in the study carried out by Halpern et al. [18]) can justify the discrepancy of the results. Only Casanova et al. [29] reported a prevalence of anemia that was lower (6.2%) than the one found in the present study.

The mechanism of the development of anemia in patients with COPD may be similar to that of other chronic diseases, in which the involvement of inflammatory response mediators in the pathogenesis of anemia has been demonstrated [28]. Our data are in agreement with this hypothesis, since CRP levels in anemic patients tended to be higher than in non anemic ones, and serum concentrations of CRP were inversely related to hemoglobin levels (r = −0349, p <0.0001). Anemic patients in the present study had more severe airflow obstruction in comparison to non-anemic patients, and they also had a significantly higher BODE index score, whereas we found no differences in CCI in our patient series, which is different from other authors [5].

It is of interest to note that the anemic subjects in our study presented a reduction in exercise capacity, expressed as 6MWD and VO2_max_, a greater degree of dyspnoea and a worse HRQL compared with non anemic patients. On the contrary, muscle strength indices were similar in both groups. Anemia has been repeatedly associated with reduced exercise capacity in patients with chronic diseases [30-35], but only 2 studies demonstrated that it is related to a reduction of VO2_max_ [17] and of distance walked at 6MWD [5] in patients with COPD. The present study confirms that anemia is a determinant of exercise capacity, regardless of the degree of airway obstruction. The mechanisms underlying the relationship between hemoglobin and exercise capacity are complex, even if the release of oxygen to the mitochondria probably plays a crucial role. When the content of arterial O_2_ is low, the gradient of diffusion of the gas from the blood to the mitochondria decreases rapidly, producing an early anaerobic metabolism [36], with consequent stimulus to ventilation. Since COPD patients have a reduced respiratory reserve, the increase in demand for ventilation may produce dyspnoea and consequently an increase in the MRCs, and a decrease in exercise capacity. It has been demonstrated that the degree of inflammation predicts decline over time of physical performance in elderly subjects [37]. In patients with COPD, a low grade systemic inflammation, as well as the inflammatory process in the lungs, has been demonstrated [38]. It is tempting to speculate that this process might play a role in the reduced exercise capacity found in our patients. However, CRP was inversely related to indices of exercise capacity in the bivariate analysis (data not shown), but it was not associated with the 6MWD or VO2_max_ in the multivariate one, when several potential confounders were taken into account. These results seem to indicate that inflammation is not a main determinant of the reduced physical performance in COPD.

To the best of our knowledge, this is the first study demonstrating that muscle strength, measured by quadriceps and handgrip strength, were not related to hemoglobin levels in COPD patients. Penninx et al. [16] found that the strength of knee extensor and of handgrip was lower in anemic than non anemic subjects [16], in non-institutionalized elderly subjects from the general population. The higher average age of the subjects in the study carried out by Penninx et al. [16], and the different sample selection, may justify the contrasting results.

As far as we know, only one study has investigated the relationship between HRQL and anemia in COPD [19]. In a survey conducted on a sample extracted from the general population and carried out by Krishnan et al. [19], it was evident that anemia is associated with a worse quality of life measured by the Short Form-36 (SF- 36) [19], in patients with moderate to severe COPD. By using a specific instrument to measure quality of life (SGRQ) in our study, we found an association between anemia, activity, impact and total scores. It is possible that a reduction in exercise capacity, related to a low hemoglobin concentration, may adversely affect the SGRQ activity score. It may also be hypothesized that anemia produces fatigue through a reduced muscle oxygenation, which in turn would lead to a decrease in physical activity, and consequently to a deterioration of psychosocial function.

An original finding of our study is the linear association between hemoglobin levels, exercise capacity and HRQL (Figure 3). These results indicate that an independent determinant of exercise capacity and HRQL is the hemoglobin concentration, even when it is not lower than the cut-off indicating the presence of anemia. It was evident from our data that a change of about two grams in hemoglobin levels is associated with an increase in the 6MWD distance of 50 meters, which is a value perceived as significant by patients [39,40]. On the other hand, the same variation of the concentrations of hemoglobin is related to a reduction of 7 points SGRQ, which corresponds to a mild to moderate improvement of the quality of life [41]. These relationships could have potential therapeutic implications, since physical performance and quality of life could be improved, not only by correcting the hemoglobin level in anemic subjects, but also by increasing its concentrations in non-anemic patients.

We emphasize that the linear relationship found in the present study is valid only for hemoglobin values between 11 and 18 mg/dL, and extrapolation to lower or higher concentrations (such as those found in severe anemia or polycythemia) is not possible. We are aware that no inference is possible from our data and that prospective studies and randomized controlled trials are warranted to test the hypothesis. However, the result of the present study support the findings of Silverberg et al. These authors demonstrated, that the correction of anemia by erythropoietin was associated with an improvement in the quality of life in hospitalized COPD patients [42].

## Conclusion

In conclusion, anemia is a frequent condition in subjects with COPD, although it seems to have a similar prevalence to that found in the general population. Even if we could not establish a causality link, our data demonstrate that anemia is associated with a reduction of exercise capacity, an increase of dyspnoea and a deterioration of the quality of life. On the contrary anemia does not seem related to muscle strength. Finally, the linear relationship between hemoglobin levels, important indices of exercise capacity, and quality of life, may provide support to pharmacological interventions in order to improve the poor health status and prognosis of patients with COPD.

## Abbreviations

HRQL: Health Related Quality of life
MRCs: Medical Research Council Dyspnoea scale
6MWD: Six minute walking distance
VO2_max_: peak of VO2 during maximal cycle ergometer test
SGRQ: St.George’s Respiratory Questionnaire
